# Prevalence of human papillomavirus in Indonesia: a population-based study in three regions

**DOI:** 10.1038/sj.bjc.6604417

**Published:** 2008-07-01

**Authors:** J N I Vet, M A de Boer, B E W M van den Akker, B Siregar, Lisnawati, S Budiningsih, D Tyasmorowati, Moestikaningsih, S Cornain, A A W Peters, G J Fleuren

**Affiliations:** 1 Department of Gynaecology and Obstetrics, Leiden University Medical center, Albinusdreef 2, P.O.Box 9600, Leiden 2300 RC, The Netherlands; 2 Department of Pathology, Leiden University Medical center, Albinusdreef 2, P.O.Box 9600, Leiden 2300 RC, The Netherlands; 3 Department of Anatomic Pathology, Faculty of Medicine University of Indonesia and National General Hospital Dr Cipto Mangunkusumo, Salemba 6, Jakarta 10430, Indonesia; 4 Department of Community Medicine, Faculty of Medicine, University of Indonesia, Pegangsaan Timur 17, Jakarta 10430, Indonesia; 5 Department of Pathology, Tasikmalaya General Hospital, Jalan Rumah Sakit 33, Tasikmalaya, Indonesia; 6 Department of Pathology, Sanglah General Hospital and Faculty of Medicine, Udayana University, Jalan Kesehatan 1 Denpasar, Bali, Indonesia

**Keywords:** HPV, prevalence, population-based, Indonesia

## Abstract

Cervical cancer is the most common cancer among women in the Indonesian population, yet little is known about the prevalence of human papillomavirus (HPV). We investigated age-specific prevalence of HPV types and possible risk factors of HPV positivity in a population-based sample of 2686 women, aged 15–70 years, in Jakarta, Tasikmalaya, and Bali, Indonesia. The overall HPV prevalence was 11.4%, age-standardized to the world standard population 11.6%. The most prevalent types found were HPV 52, HPV 16, HPV 18, and HPV 39, respectively, 23.2, 18.0, 16.1, and 11.8% of the high-risk HPV types. In 20.7% of infections, multiple types were involved. Different age-specific prevalence patterns were seen: overall high in Jakarta, and in Tasikmalaya, and declining with age in Bali. The number of marriages was most associated with HPV positivity (OR 1.81 95% CI 1.31–2.51)). Remarkably, in Indonesia HPV 16 and HPV 18 are equally common in the general population, as they are in cervical cancer. HPV 52 was the most prevalent type in the general population, suggesting that this type should be included when prophylactic HPV vaccination is introduced in Indonesia.

Cervical cancer is the most common cancer in women in Indonesia, as in most developing countries (Tjindarbumi and Mangunkusumo, 2002; Parkin *et al*, 2005). From hospital-based data, it accounts for 28.6% of female cancers in Indonesia (Tjindarbumi and Mangunkusumo, 2002).

Human papillomavirus (HPV) prevalence, age-specific prevalence, and type of distribution differ substantially between populations (Clifford *et al*, 2005; Franceschi *et al*, 2006) and HPV 18 has a greater role in cervical cancer in Indonesia than in the rest of the world. HPV 18 was found as frequently as HPV 16 in cervical cancer (Schellekens *et al*, 2004), or even more frequently than HPV 16 (Bosch *et al*, 1995). A small hospital-based case–control study conducted in Jakarta also found a high prevalence of HPV 18 in controls (de Boer *et al*, 2006). In view of the lack of population-based relevant data, we report here the age-specific prevalence data for HPV among women in Jakarta and Tasikmalaya on the island of Java and among women on the island of Bali, and assess possible risk factors in the aetiology of cervical cancer.

## Material and methods

This population-based study was conducted as part of a screening project for cervical cancer in Jakarta and Tasikmalaya on the island of Java and different regions on the island of Bali in Indonesia between October 2004 and February 2006. Women were excluded if they were virgin, pregnant, had undergone a hysterectomy, or had previous cervical cancer. During our study it appeared that only married, divorced or widowed women participated in screening. Women who had never been married would strictly still be virgins because sexual intercourse before marriage is not allowed following Indonesia's cultural and religious rules.

To reach women, in each region a collaboration was set up with the Pembinaan Kesejahteraan Keluarga (PKK), the national Indonesian Family Welfare Organization. The smallest branches of this governmental women's movement organise activities at the village level. The attempt was made to invite all women aged 20–65 years in the selected villages and to screen at least 80% of these. Members of this local PKK invited participation by visiting women at home and informing them about risk factors, prevention, early detection, and treatment of premalignant cervical cancer lesions. Participants were individually counselled by public health nurses and their informed consent obtained; all were interviewed about socio-demographic, reproductive and cervical cancer risk factors.

A total of 20 834 women, aged 12–70, were screened: 6274 from Jakarta, 8007 from Tasikmalaya, and 6553 from Bali. For the HPV typing, a random age-stratified sample of the participants was taken for each region by categorising the women in 11 5-year age groups: ⩽19, 20–24, 25–29, 30–34, 35–39, 40–44, 45–49, 50–54, 55–59, 60–64, and ⩾65 years. In each age group, 100 randomly selected samples were used for HPV analysis. As the youngest and oldest groups were under-represented (<100 women), the total samples was less than 1100 per region. For Jakarta 915 were selected, for Tasikmalaya 975, and for Bali 950 samples.

Participants underwent a visual inspection of the cervix and a smear obtained using a wooden Ayre spatula for the ectocervix and a cytobrush for the endocervix. Slides were immediately fixed with ethanol and further processed for diagnosing by cytoscreeners. The exfoliated cells remaining on the spatula and brush were suspended in 25 ml of phosphate-buffered saline in a 50 ml Falcon tube. The tubes were centrifuged at 3000 **g** for 5 min. The supernatant was removed, and the cell pellet was re-suspended in 1 ml of phosphate-buffered saline and transferred to a 1.5 ml Eppendorf tube with a safety lock. All tubes were directly frozen and stored in a −20°C freezer and shipped on dry ice to the Department of Pathology, Leiden University Medical Center, the Netherlands.

Furthermore, all women underwent visual inspection with acetic acid and those women with acetowhite lesions and/or cytological abnormalities were treated with cryotherapy. In cases of suspected cervical cancer the women were referred to the collaborating university hospitals.

To test the quality of DNA obtained from exfoliated cells, a polymerase chain reaction on the human genomic *β*-globin gene was performed. HPV DNA detection and genotyping was performed, amplified using the SPF10 primer and HPV amplimers tested on agarose gels (Kleter *et al*, 1999).

The genotyping of positive products was performed using an INNO-line probe assay prototype research genotyping assay (Innogenetics, Ghent, Belgium which detected the following 25 types: HPV 6, 11, 16, 18, 31, 33, 34, 35, 39, 40, 42, 43, 44, 45, 51, 52, 53, 54, 56, 58, 59, 66, 68, 70, and 74. High-risk types were identified using the HPV well known categories (Longuet *et al*, 1996; Munoz *et al*, 2003).

### Statistical analysis

Statistical analysis was performed using SPSS software (SPSS, version 12, SPSS Inc., Chicago, IL, USA). For each region odds ratios (ORs) and 95% confidence intervals (CIs) using unconditional multiple logistic regression adjusted for age were calculated to estimate the association between HPV infections and risk factors adjusted for age. To test for linear trend for odds ratios, a *χ*^2^ linear test was calculated. Age standardization of rates for ages 15–70 was calculated using the world standard (Segi, 1960).

## Results

Of the 2840 patients, 114 cellular samples were missing (26 Jakarta, 48 Tasikmalaya, 40 Bali). Of the remaining 2726, 40 samples were excluded because of a negative *β*-globin test (9 from Jakarta, 8 from Tasikmalaya, and 23 from Bali), of the final study group (2686 samples: 880 Jakarta, 919 Tasikmalaya, 887 from Bali). 91.2% had never been screened before, 80.7% from Jakarta, 97.4% Tasikmalaya and 95.6% Bali. Based on cytology, five of 880 women in Jakarta were diagnosed with cervical cancer, four of the 919 in Tasikmalaya and three of the 887 in Bali. Based on visual inspection with acetic acid, there were also two suspected cases in Tasikmalaya. In 13 of the 14 cases, the diagnoses were histologically confirmed and in the other the woman repeatedly refused follow up.

Overall, 305 samples (11.4%) were HPV positive (11.6% when world age-standardized), in Jakarta 122 samples (13.9% standardized 13.2%) were HPV positive, in Tasikmalaya 81 (8.8% standardized 9.0%), and in Bali 102 samples (11.5% standardized 12.1%). In total, in 211 samples (7.9%) high-risk HPV type was detected: 77 (8.9%) in Jakarta; 62 (6.7%) in Tasikmalaya and 72 (8.1%) in Bali. Forty-six samples (22 from Jakarta, 8 from Tasikmalaya, and 16 from Bali) were positive by the SPF10 primer set but not for HPV types represented in the line probe assay; these indicated as HPV X could not be categorised as cancer-associated or non-cancer-associated types.

Twenty-four different HPV types were detected, the commonest in descending order of prevalence being HPV 52, 16, and 18. This most differed by region, as shown in Table 1. Multiple HPV types were detected in 63 samples (2.3% overall, or 20.7% of all positive samples): in Jakarta in 22 (2.5 or 18.0%), in Tasikmalaya in 15 (1.6 or 18.5%), and in Bali in 26 (2.9 or 25.5%).

In the 14 cervical cancer cases, single HPV 52 infection was detected in three and HPV 18 was also detected in three cases including one multiple infection with HPV 6. HPV 16 was detected two times, HPV 31, HPV 33 and HPV 39, HPV42 and HPV 45 were detected once. In one cancer case, HPV could not be detected.

**Figure 1:**
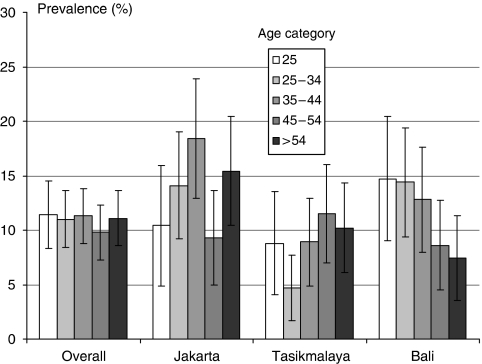
Age-specific prevalence of cervical human papillomavirus (HPV) DNA in percentages with 95% CI (Overall, Jakarta, Tasikmalaya and Bali, Indonesia).

The age-specific prevalence overall, and by region is shown in Figure 1. The overall age-specific prevalence in Indonesia was high in all ages (⩾9.8%), and ranged from 9.8 to 13.3%. In Jakarta, the overall prevalence was high (⩾9.3%), with two peaks: one of 18.4% in the 35–44 year age group and a second, of 15.4%, in women older than 54 years. The age-specific prevalence in Tasikmalaya peaked at 8.8% in the youngest age group of <25 years, declined to 4.7% in the 25–34 year group, and formed a second peak at 11.5% in the 45–54 year group. In Bali, HPV prevalence declined with age, from 14.7% in the <25 year group to 7.4% in the >54 year group, the trend in this decline was significant (*P*<0.01).

### Risk factors for HPV infection

The association between HPV infection and characteristics of the studied women overall and by region after adjustment for age are shown in Table 2. Overall, being screened before was associated with a higher HPV positivity than never being screened before (OR 1.67, 95% CI 1.13–2.44) as was having had more than one partner than women having had one partner (OR 1.81, 95% CI 1.31–2.51). In Jakarta, HPV positivity was inversely associated with daily income when women having ⩾US $3 per day were compared with those with ⩽US $1 per day (OR 0.51, 95% CI 0.28–0.92, with a significant trend of *P*=0.02), while having had more than one partner was associated with a higher HPV positivity than one partner (OR 2.66, 95% CI 1.64–4.33). In Tasikmalaya, the few divorced women had an OR of 6.0 (95% CI 1.39–25.91) with HPV positivity compared with married women. Although not significant, it seemed that nulliparous women were more often HPV positive (OR 5.35, 95% CI 0.92–31.08) than women with 1–2 children. In Bali, being 55 years or older was inversely associated with positivity (OR 0.46, 95% CI 0.23–0.96). In contrast with Jakarta, in Bali higher daily income was associated with higher positivity (OR 1.91, 95% CI 1.08–3.38, with a trend of *P*=0.03). In all three regions, HPV positivity was unrelated to having had a previous Pap smear, education level, smoking, the number of miscarriages, age at menarche, age at marriage, or first pregnancy.

## Discussion

Among a mainly unscreened population of women in Indonesia, we found an intermediate overall prevalence of HPV, highest types 52, 16, and 18, with a different age specific pattern in the three regions.

Clifford *et al* defined age-standardized HPV prevalence rates as ‘low’, ‘intermediate’, and ‘high’. Using a previous classification, the overall prevalence found (11.3%) was intermediate (Clifford *et al*, 2005), comparable to other Asian countries like Thailand (Lampang, 9.1%), Vietnam (Ho Chi Minh City, 10.6%), India (17.7%), and with South American countries such as Chile (14.0%) and Mexico (14.5%) (Lazcano-Ponce *et al*, 2001; Pham *et al*, 2003; Sukvirach *et al*, 2003; Ferreccio *et al*, 2004; Franceschi *et al*, 2005). Cervical cancer incidence is related to HPV prevalence in the region and the presence of organised screening, among other factors (Bosch and de Sanjose, 2003) and that estimated for Indonesia, a country without organised screening (at least 30 per 100 000 women per year) is consistent with recorded HPV (Schellekens *et al*, 2004; Parkin *et al*, 2005) prevalence.

Areas of high HPV prevalence and with no decline in older age groups all have high incidence and mortality and very low income levels (Franceschi *et al*, 2006), as is true for Jakarta and Tasikmalaya.

In contrast, in Bali a significant decline in HPV prevalence with increasing age was seen, as in some western countries, in Korea (Franceschi *et al*, 2006) and among Hindu women in India (Duttagupta *et al*, 2004). In the latter, the reproductive period (and thus active sexual life) of Hindu women mostly ends by the age of 30–35 years, which could explain the decreasing prevalence observed. Genetic factors in the susceptibility to HPV infection in older age could not be excluded. Unfortunately, we lack such information for Hindu women in our study.

Two potential selection biases could have occurred: we attempted to screen at least 80% of all women aged 20–65 in the villages who were visited by the screening programme. All women were informed at their homes but only the women who actually answered the call to participate were included. The percentage of targeted women included was only available for Bali, (83.7%). Because the method of selection was similar in all regions, and because all households in an area were visited, we assume that participation is similar to that in Bali. The major reasons for non-participation were having to work, sickness or anxiety. The second bias concerns the fact that culturally and religiously, sexual intercourse is not allowed before marriage in Indonesia. For this reason a few questions were believed to be inappropriate and thought not to be answered honestly. Instead we asked acceptable questions: ‘*age of first marriage’* and ‘*number of marriages*’. We did not perform gynaecological examination on unmarried women who would strictly still be virgin; all our screened women were married, divorced or widowed.

HPV 52 was the most prevalent type in Jakarta and Bali, and the second most prevalent type (after 18) in Tasikmalaya accounting, respectively, for 23.1, 20.1, and 23.4% of the high-risk positive samples. Worldwide, high prevalence of HPV 52 is also reported from China, Taiwan, and Costa Rica (Herrero *et al*, 2005; Jeng *et al*, 2005; Dai *et al*, 2006). It was also detected, in 21.4% (three of 14) of the cervical cancers as it was in 14% of cases in another study (Schellekens *et al*, 2004). Clifford *et al* (2003) also identified HPV 52 more frequently in cervical cancer in Asia than in other parts of the world. Adding HPV 52, to types 16 and 18, in a prophylactic HPV vaccine when introduced in Indonesia would therefore seem appropriate.

HPV 16 prevalence in this population was comparable to that in most parts of the world (Clifford *et al*, 2005), though that of HPV 18 was higher than regions with comparable overall HPV prevalence (Lazcano-Ponce *et al*, 2001; Pham *et al*, 2003; Sukvirach *et al*, 2003; Ferreccio *et al*, 2004; Franceschi *et al*, 2005). HPV 18 accounted for a significant population of high-risk HPV-positive samples: 10.3, 22.6, and 15.6%, respectively, for Jakarta, Tasikmalaya, and Bali. In most countries, HPV 16 is by far the most prevalent type in cervical carcinoma and in the general population, followed by HPV 18 (Clifford *et al*, 2003, 2005). Although we found small inter-regional differences (see Table 1), HPV 18 was as common as 16, reflecting their predominant roles in cervical cancer in Indonesia (Bosch *et al*, 1995; Schellekens *et al*, 2004). HPV 39 was prevalent in the general population in this study, but it is rare in cervical carcinomas in Indonesia and other parts of Asia (Clifford *et al*, 2003; Schellekens *et al*, 2004).

As expected, a history of more than one sexual partner was associated with HPV positivity (Vaccarella *et al*, 2006a, 2006b). A small group of divorced women in Tasikmalaya and women with high daily income in Bali were associated with HPV positivity, probably reflecting their sexual behaviour and that of their partners (Vaccarella *et al*, 2006a). Unfortunately, little relevant information is available because sexuality is still a taboo subject in Indonesia; more research could be revealing. Overall, previous screening was associated with higher HPV positivity. Possibly, reflecting earlier symptoms.

In conclusion, in Indonesia HPV 16 and 18 are equally common in the general population, as they are in cervical cancer. HPV 52 was the most prevalent type, suggesting that it should also be included in the prophylactic HPV vaccine when introduced in Indonesia.

## Figures and Tables

**Table 1:** Prevalence of type-specific cervical human papilloma infection types among 2686 women in Indonesia

	**Jakarta (880)**				**Tasikmalaya (919)**				**Bali (887)**			
**HPV type^a^**	**Single**	**Multiple**	**Total^b^ (%)**	**HPV+^c^ (%)**	**Single**	**Multiple**	**Total^b^ (%)**	**HPV+^c^ (%)**	**Single**	**Multiple**	**Total^b^ (%)**	**HPV+^c^ (%)**
Negative			758 (86.1)				838 (91.2)				785 (88.5)	
Positive	100	22	122 (13.9)		66	15	81 (8.8)		76	26	102 (11.5)	
High risk	56	22	78 (8.9)	63.9	47	15	62 (6.7)	76.5	52	25	77 (8.9)	75.4
Low risk	22	—	22 (2.5)	18.0	11	—	11 (1.2)	13.6	8	1	9 (1.0)	8.8
X	22	—	22 (2.5)	18.0	8	—	8 (0.9)	9.9	16	—	16 (1.8)	15.7
												
*High risk*												
16	10	3	13 (1.5)	10.7	5	5	10 (1.1)	12.3	8	7	15 (1.7)	15.0
18	5	3	8 (0.9)	6.6	10	4	14 (1.5)	17.3	7	5	12 (1.4)	12.0
31	—	1	1 (0.1)	0.8	1	—	1 (0.1)	1.2	2	1	3 (0.3)	3.0
33	1	1	2 (0.2)	1.6	—	1	1 (0.1)	1.2	3	3	6 (0.7)	5.9
35	3	—	3 (0.3)	2.5	2	1	3 (0.3)	3.7	2	—	2 (0.2)	2.0
39	7	4	11 (1.3)	9.0	7	1	8 (0.9)	9.9	3	3	6 (0.7)	6.0
45	—	4	4 (0.5)	3.3	—	1	1 (0.1)	1.2	2	4	6 (0.7)	6.0
51	6	4	10 (1.1)	8.2	2	2	4 (0.4)	4.9	7	1	8 (0.9)	8.0
52	13	5	18 (2.0)	14.8	8	5	13 (1.4)	16	10	8	18 (2.0)	18.0
53	3	2	5 (0.6)	4.1	3	3	6 (0.7)	7.4	1	—	7 (0.8)	7.0
56	6	1	7 (0.8)	5.7	4	2	6 (0.7)	7.4	1	5	6 (0.7)	6.0
58	1	—	1 (0.1)	0.8	2	—	2 (0.2)	2.5	—	2	2 (0.2)	2.0
59	1	1	2 (0.2)	1.6	—	—	—		—	—	—	
66	—	1	1 (0.1)	0.8	1	—	1 (0.1)	1.2	2	2	4 (0.5)	4.0
68	—	3	3 (0.3)	2.5	2	—	2 (0.2)	2.5	4	1	5 (0.6)	5.0
												
*Low risk*												
6	5	3	8 (0.9)	6.6	—	2	2 (0.2)	2.5	4	2	6 (0.7)	6.0
11	—	—	—		—	—	—		—	1	1 (0.1)	1.0
40	—	—	—		—	1	1 (0.1)	1.2	1	1	2 (0.2)	2.0
42	—	—	—		1	—	1 (0.1)	1.2	—	—	—	
43	2	—	2 (0.2)	1.6	1	2	3 (0.3)	3.7	—	2	2 (0.2)	2.0
44	1	2	3 (0.3)	2.5	1	1	2 (0.2)	2.5	1	2	3 (0.3)	3.0
54	4	2	6 (0.7)	4.9	2	1	3 (0.3)	3.7	—	2	2 (0.2)	2.0
70	4	4	8 (0.9)	6.6	5	2	7 (0.8)	8.6	2	4	6 (0.7)	6.0
74	6	1	7 (0.8)	5.7	1	—	1 (0.1)	1.2	—	2	2 (0.2)	2.0

a The same woman can be counted more than once because of multiple infections.

b Percentage over all women.

c Percentage over HPV-positive cases.

**Table 2:** ORs for HPV detection and corresponding 95% CI

	**Overall**			**Jakarta**			**Tasikmalaya**			**Bali**		
	**HPV**	**HPV**		**HPV**	**HPV**		**HPV**	**HPV**		**HPV**	**HPV**	
	**neg**	**pos**	**OR^a^ (95% CI)**	**neg**	**pos**	**OR^a^ (95% CI)**	**neg**	**pos**	**OR^a^ (95% CI)**	**neg**	**pos**	**OR^a^ (95% CI)**
*Age*												
<25	365	47	1	112	13	1	125	12	1	128	22	1
25–34	509	63	0.96 (0.64–1.44)	165	27	1.41 (0.70–2.85)	183	9	0.51 (0.21–1.25)	161	27	0.98 (0.53–1.79)
35–44	494	76	1.20 (0.81–1.76)	155	35	1.95 (0.98–3.85)	175	17	1.01 (0.47–2.19)	164	24	0.85 (0.46–1.59)
45–54	495	54	0.85 (0.56–1.28)	156	16	0.88 (0.41–1.91)	170	22	1.35 (0.64–2.83)	169	16	0.55 (0.28–1.09)
⩾55	518	65	0.97 (0.65–1.45)	170	31	1.57 (0.79–3.13)	185	21	1.18 (0.56–2.49)	163	13	0.46 (0.23–0.96)
Trend	0.48			0.63			0.11			0.01		
												
*Screened before*												
No	2186	267	1	615	95	1	817	78	1	754	94	1
Yes	187	37	1.67 (1.13–2.44)	141	27	1.25 (0.76–2.05)	19	3	1.43 (0.41–5.00)	27	7	1.98 (0.83–4.75)
												
*Salary*												
<1$ per day	840	104	1	99	23	1	371	42	1	370	39	1
1–3$ per day	861	109	1.01 (0.76–1.35)	353	61	0.77 (0.45–1.30)	224	13	0.53 (0.27–1.01)	284	35	1.11 (0.68–1.81)
>3$ per day	583	75	1.03 (0.75–1.42)	257	31	0.51 (0.28–0.92)	214	21	0.82 (0.47–1.45)	112	23	1.91 (1.08–3.38)
Trend	0.80			0.02			0.41			0.03		
												
*Marital status*												
Married	2260	287	1	683	111	1	795	74	1	783	102	1
Divorced	10	4	2.96 (0.92–9.55)	5	1	1.12 (0.13–9.85)	5	3	6.0 (1.38–25.91)	0	0	0
Widowed	109	14	1.07 (0.59–1.93)	70	10	0.85 (0.40–1.80)	39	4	0.9 (0.31–2.64)	0	0	0
												
*Age at marriage*												
<16	287	42	1	96	21	1	144	16	1	47	5	1
16–18	449	56	0.85 (0.55–1.30)	218	33	0.70 (0.38–1.28)	325	31	0.92 (0.48–1.74)	217	34	1.49 (0.55–4.03)
19–21	535	62	0.79 (0.52–1.20)	242	32	0.60 (0.33–1.10)	224	21	0.96 (0.48–1.92)	328	37	1.21 (0.44–3.28)
>21	1104	145	0.89 (0.62–1.29)	202	36	0.79 (0.43–1.45)	144	13	0.92 (0.42–2.02)	188	26	1.37 (0.49–3.87)
Trend	0.67			0.64			0.61			0.84		
												
*Number of marriages*												
1	2112	250	1	675	92	1	687	61	1	750	97	1
>1	261	55	1.81 (1.31–2.51)	82	30	2.66 (1.64–4.33)	149	20	1.40 (0.81–2.43)	30	5	1.51 (0.56–4.06)
												
*Parity*												
0	22	3	1.03 (0.30–3.50)	9	1	0.76 (0.09–6.23)	4	2	5.34 (0.92–31.06)	9	0	0
1/2	1063	138	1	302	41	1	333	33	1	428	64	1
3/4	719	87	0.91 (0.67–1.25)	233	47	1.43 (0.84–2.41)	272	20	0.60 (0.31–1.15)	214	20	0.71 (0.40–1.28)
⩾5	455	58	0.99 (0.66–1.47)	172	23	0.96 (0.50–1.85)	175	25	1.09 (0.54–2.20)	108	10	0.82 (0.35–1.90)
Trend	0.83			0.73			0.51			0.11		

OR=odds ratio, CI=confidence interval.

Figures do not add up to the total because of missing values.

a Adjusted for age.
